# Immunomodulatory roles of the HLA family and B2M in atherosclerosis: Diagnostic biomarkers and thrombosis risk

**DOI:** 10.1097/MD.0000000000050480

**Published:** 2026-09-04

**Authors:** Ke Ma, Jingping Ge

**Affiliations:** a Women’s Hospital of Nanjing Medical University (Nanjing Women and Children’s Healthcare Hospital), Nanjing, Jiangsu, P.R. China; b Department of Interventional Radiology, Nanjing First Hospital, Nanjing Medical University, Nanjing, Jiangsu, P.R. China.

**Keywords:** atherosclerosis, B2M, HLA family, immune microenvironment, thrombosis

## Abstract

Atherosclerosis (AS), a chronic inflammatory disease driven by lipid accumulation and immune dysregulation, predisposes to acute cardiovascular events via plaque rupture and thrombosis. The molecular mechanisms linking immune microenvironment imbalance to plaque instability remain incompletely defined. Utilizing the GSE100927 dataset (69 AS patients vs 35 controls), we identified differentially expressed genes (DEGs) and constructed co-expression networks via weighted gene co-expression network analysis. Hub genes were intersected with DEGs to determine core genes. Immune cell infiltration was quantified using Cell-type Identification By Estimating Relative Subsets Of RNA Transcripts, and correlations between core genes and immune subsets were assessed. Diagnostic potential was evaluated by receiver operating characteristic (ROC) analysis. We identified 2596 DEGs (1575 up-/1021 downregulated) in AS tissues. Weighted gene co-expression network analysis revealed 3 disease-associated modules, yielding 54 hub genes. Intersection with DEGs identified 12 core genes, including HLA-G, HLA-A, HLA-C, and beta-2-microglobulin (B2M). These genes were enriched in “major histocompatibility complex class Ib-mediated antigen presentation” and significantly upregulated in AS (*P* < .001). Immune profiling showed elevated M0 macrophages and reduced resting CD4^+^ memory T cells in AS (*P* < .0001). Expression of HLA-G, HLA-A, HLA-C, and B2M positively correlated with M0 macrophages and negatively with CD4^+^ T cells (*P* < .001). ROC analysis within the GSE100927 dataset demonstrated high diagnostic accuracy for distinguishing AS samples from controls (HLA-G/A/C area under the ROC curve = 0.94; B2M area under the ROC curve = 0.87). HLA-G, HLA-A, HLA-C, and B2M modulate macrophage and T cell functions, driving immune dysregulation in AS. This immune imbalance is associated with plaque instability and may contribute to thrombosis risk, highlighting the potential of these molecules as diagnostic biomarkers and therapeutic targets.

## 1. Introduction

Atherosclerosis (AS) is a chronic, progressive vascular disease characterized by lipid accumulation within the arterial wall, persistent inflammation, and the formation of atherosclerotic plaques.^[1]^ AS-related coronary heart disease and stroke are among the leading causes of mortality worldwide.^[2]^ Cardiovascular disease accounts for approximately 17.9 million deaths annually, representing nearly 32% of all global deaths, according to the World Health Organization.^[3]^ Plaque rupture followed by thrombosis formation constitutes a pivotal pathophysiological mechanism in the onset of acute cardiovascular events, such as myocardial infarction and ischemic stroke.^[4]^ Therefore, elucidating the pathogenesis of AS and its association with thrombosis is of significant clinical importance for developing targeted prevention and treatment strategies. Endothelial dysfunction is considered a critical initiating event in the pathogenesis of AS. Following endothelial injury, low-density lipoprotein (LDL) infiltrates the subendothelial space and undergoes oxidative modification. This oxidative stress promotes the recruitment of circulating monocytes, which subsequently differentiate into macrophages. Through scavenger receptor-mediated phagocytosis of oxidized LDL (ox-LDL), these macrophages transform into foam cells, thereby establishing the lipid core that characterizes early atherosclerotic lesions.^[5]^ As AS progresses, foam cell apoptosis, the release of inflammatory factors (such as tumor necrosis factor-alpha [TNF-α] and interleukin-1), and the proliferation and migration of vascular smooth muscle cells collectively contribute to the formation of fibrous plaques.^[6]^ In recent years, studies have demonstrated that the instability of atherosclerotic plaques is closely associated with thrombus formation. As plaques enlarge, they develop a fibrous cap that initially stabilizes the lesion but may become vulnerable over time. Unstable plaques are prone to rupture due to thin fibrous caps, enlarged lipid cores, and the infiltration of activated immune cells. The exposure of procoagulant substances upon rupture triggers platelet activation and initiates the coagulation cascade, ultimately resulting in thrombus formation.^[7]^

Although significant progress has been made in the study of AS, its molecular mechanism has not yet been fully elucidated. Traditionally, dysregulated lipid metabolism has been regarded as the initiating factor in AS. However, accumulating evidence suggests that inflammatory responses driven by immune microenvironment imbalances represent the central mechanism underlying plaque instability and thrombus formation. For example, in the pathological process of AS, macrophages differentiate into foam cells by phagocytosis of lipids, thereby forming the lipid core of early atherosclerotic lesions. Additionally, dysfunction of T cell subsets, such as CD4^+^ memory T cells, exacerbates local inflammatory responses and contributes to plaque instability, while also releasing procoagulant factors that directly promote thrombus formation.^[8]^ Moreover, major histocompatibility complex (MHC) molecules directly influence the progression and stability of atherosclerotic lesions by mediating the presentation of AS-related antigens, such as ox-LDL, by antigen-presenting cells to T cells. This interaction regulates the balance between pathogenic T cell subsets, such as T helper 1 (Th1) cells, and regulatory T cells (Tregs), thereby modulating local immune responses within the plaque microenvironment.^[9]^ The above results suggest that exploring the expression patterns and immune cell infiltration characteristics of immune-related genes in AS may provide a new perspective for revealing disease mechanisms and identifying thrombus risk markers.

Currently, advances in high-throughput sequencing technologies offer powerful tools for the systematic analysis of the molecular characteristics of AS. Screening differentially expressed genes (DEGs) through gene expression profiling, combined with weighted gene co-expression network analysis (WGCNA) to identify core regulatory genes (hub genes), has become an effective strategy for mining key molecules in diseases.^[10]^ At the same time, immune infiltration analysis can quantify the differences in the composition of immune cells in the sample, providing a basis for the function of associated genes and the immune microenvironment.^[11]^ However, the specific mechanisms by which core genes in AS influence disease progression and thrombosis through the regulation of immune cell functions remain to be elucidated. Therefore, this study utilized the GSE100927 dataset from the gene expression omnibus (GEO) database to perform a comprehensive analysis. AS-related DEGs and hub genes were identified through bioinformatics approaches, and their functional enrichment characteristics were systematically analyzed. In parallel, immune infiltration analysis was conducted to investigate alterations in immune cell composition in patients with AS, as well as to assess the correlations between core genes and specific immune cell subsets. The aim of this study was to elucidate the potential molecular mechanisms underlying the development of AS, identify key genes associated with immune regulation and thrombosis, and provide a theoretical foundation for the early diagnosis and targeted therapy of AS and its thrombotic complications.

## 2. Materials and methods

### 2.1. Data acquisition and pre-processing

In this study, we utilized the GEO database (https://www.ncbi.nlm.nih.gov/geo/) to identify relevant human case-control transcriptomic datasets. A systematic search was conducted using “atherosclerosis” as a keyword, focusing on experimental studies published within the past 5 years. Following a comprehensive screening process, the GSE100927 dataset was selected for downstream analysis due to its data quality and relevance. The dataset was retrieved using the GEOquery package in R, which facilitated the extraction of the original gene expression matrix and associated sample metadata. GSE100927 comprises gene expression profiles from 104 samples, including 69 AS patients and 35 healthy controls. To ensure data consistency and reliability, the raw expression matrix underwent rigorous preprocessing, which involved standardization, batch effect correction, and normalization procedures. These steps were essential to mitigate technical variability and prepare the data for subsequent differential expression and functional enrichment analyses.

### 2.2. Differential expression analysis and functional enrichment analysis

To identify genes associated with AS, we utilized the limma package in R to screen for DEGs between the processed AS and control group samples. The distribution of DEGs was visualized using the ggplot2 package to generate a volcano plot. Subsequently, the clusterProfiler package was employed to perform gene ontology (GO) functional enrichment analysis and Kyoto Encyclopedia of Genes and Genomes pathway analysis to elucidate the biological functions and pathways associated with the identified DEGs.

### 2.3. WGCNA

To investigate the functional modules of genes associated with AS, we constructed gene co-expression networks using the expression profile data of all genes from the GSE100927 dataset. WGCNA was employed to identify gene modules with similar expression patterns. Module eigengenes were calculated and correlated with clinical traits using Pearson correlation analysis. Modules significantly associated with AS were selected, and hub genes within these modules were identified for further analysis.

### 2.4. Core gene set identification and functional enrichment analysis

A core gene set was established by integrating overlapping DEGs and hub genes. GO enrichment analysis of these overlapping genes was subsequently conducted using the clusterProfiler R package.

### 2.5. Immunoinfiltration analysis

The gene expression matrix was uploaded to the Cell-type Identification By Estimating Relative Subsets Of RNA Transcripts online platform (https://cibersortx.stanford.edu) for immune cell deconvolution, utilizing the built-in leukocyte signature matrix to estimate the relative proportions of 22 immune cell types in samples from both AS patients and healthy controls. The resulting immune cell composition profiles were visualized using the ggplot2 and ComplexHeatmap R packages. Subsequently, intergroup differences in immune cell infiltration were statistically evaluated to characterize the immune microenvironmental features associated with AS.

### 2.6. Correlation and diagnostic validation of core genes in relation to immune infiltration

To further elucidate the relationship between core gene expression and immune microenvironment characteristics, Spearman rank correlation analysis was performed to assess the associations between core gene expression levels and immune cell proportions. The linkET R package was utilized to visualize the correlation network, while the false discovery rate (FDR) method was applied to adjust for multiple testing. Differences in core gene expression between the AS and healthy control groups were illustrated using violin plots. Additionally, the diagnostic performance of the core genes for AS was evaluated by calculating the area under the receiver operating characteristic (ROC) curve (AUC) using the pROC R package.

### 2.7. Statistical methods and thresholds for differential expression, network, and diagnostic analyses

All statistical analyses were performed using R software (version 4.2.0; R Core Team, R Foundation for Statistical Computing), with data visualization performed using the ggplot2 and ComplexHeatmap packages. The specific statistical methods and significance thresholds applied in this study are as follows. First, differential expression analysis (DEG screening): DEGs were identified using the limma package, with thresholds set at |log2 fold change (log2FC)| > 0.5 and an FDR < 0.05. Second, WGCNA: Pearson correlation coefficients were calculated to correlate module eigengenes with disease status. Modules with significant correlations (*P* < 1e−5) were considered, and hub genes within these modules were identified based on gene significance > 0.4 and module membership > 0.5. Third, immune infiltration analysis: differences in immune cell proportions between groups were evaluated using the Wilcoxon rank-sum test, with statistical significance defined as *P* < .05. Fourth, correlation analysis: Spearman rank correlation analysis was performed to assess the associations between gene expression levels and immune cell proportions. Multiple testing corrections were applied using the FDR method. Fifth, expression differences of core genes: the independent samples *t*-test was used to compare core gene expression levels between the AS and healthy control groups. A significance threshold of *P* < .001 was applied. Sixth, diagnostic performance evaluation: the AUC was calculated using the pROC R package to evaluate the diagnostic performance of core genes for AS.

### 2.8. Clinical characteristics of the cohort

The clinical baseline information available for the GSE100927 cohort is summarized herein. The cohort consisted of 69 AS patients and 35 control subjects. The sex distribution was not significantly different between the 2 groups (*P* > .05, chi-square test). The atherosclerotic samples were derived from 3 vascular beds: the carotid artery (n = 29), femoral artery (n = 26), and infra-popliteal artery (n = 14). The control samples were also annotated with these same anatomical labels (carotid: n = 12; femoral: n = 12; infra-popliteal: n = 11), suggesting they represent non-diseased tissue segments from corresponding anatomical locations (Table 1). The complete clinical metadata is provided in Table S1, Supplemental Digital Content 1. Detailed patient-level data, such as age, were not available in the dataset metadata.

**Table 1:** Clinical characteristics of the study cohort from GSE100927.

Characteristic	AS Group (n = 69)	Control Group (n = 35)	*P* value
Sex, n (%)			> .05
Male	57 (82.6)	28 (80.0)	
Female	12 (17.4)	7 (20.0)	
Tissue source, n			
Carotid artery	29	12	
Femoral artery	26	12	
Infra-popliteal artery	14	11	

AS = atherosclerosis, n = number of participants.

## 3. Results

### 3.1. Differential transcriptomic profiling of AS

**Figure 1.**
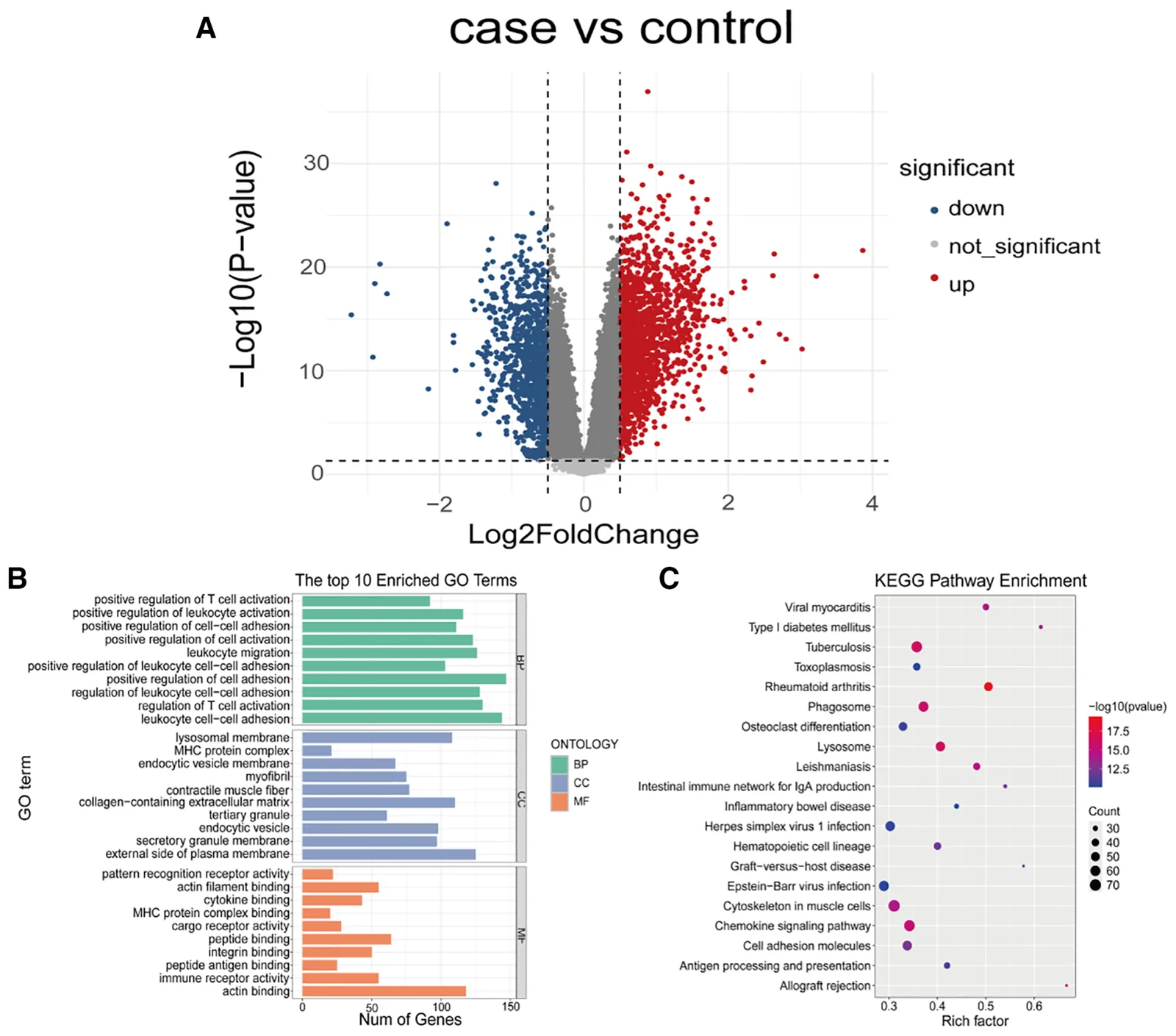
Differential gene expression and functional enrichment analysis in AS. (A) Volcano plot of DEGs between AS patients and healthy controls. Red dots represent significantly upregulated genes (n = 1575, |logFC| > 0.5, FDR < 0.05), blue dots indicate downregulated genes (n = 1021), and gray dots denote nonsignificant genes. Dotted lines indicate logFC thresholds (± 0.5). (B) GO functional enrichment analysis of DEGs, categorized into BP (green), CC (blue), and MF (orange). (C) KEGG pathway enrichment (top 20) of DEGs. Bubble size reflects the number of enriched genes; color gradient represents statistical significance (−log10 FDR), with darker red indicating higher enrichment significance. AS = atherosclerosis, BP = biological processes, CC = cellular components, DEG = differentially expressed genes, FDR = false discovery rate, GO = gene ontology, KEGG = Kyoto Encyclopedia of Genes and Genomes, MF = molecular functions, MHC = major histocompatibility complex.

Differential expression analysis identified a total of 2596 DEGs, of which 1575 genes were upregulated and 1021 genes were downregulated in the AS group (Fig. 1A). These findings indicate extensive transcriptomic alterations in the arterial tissues of AS patients, with a notably higher number of upregulated genes, suggesting widespread gene activation during AS pathogenesis.

To elucidate the biological function of these differential genes, functional enrichment analyses were subsequently performed. GO enrichment analysis revealed significant enrichment of immune-related biological processes (BP), including “intercellular adhesion” and “leukocyte activation” (Fig. 1B).

Additionally, Kyoto Encyclopedia of Genes and Genomes pathway analysis demonstrated that key immune regulatory pathways, such as “cell adhesion molecules,” “antigen processing and presentation,” and the “chemokine signaling pathway,” were significantly activated (Fig. 1C). Collectively, these results suggest that dysregulation of immune-associated BP and pathways constitutes a critical molecular mechanism in AS progression. The activation of these immune responses may enhance intraplaque inflammation, thereby increasing the risk of plaque rupture and thrombosis.

### 3.2. Co-expression network analysis identifies disease-associated gene modules

**Figure 2.**
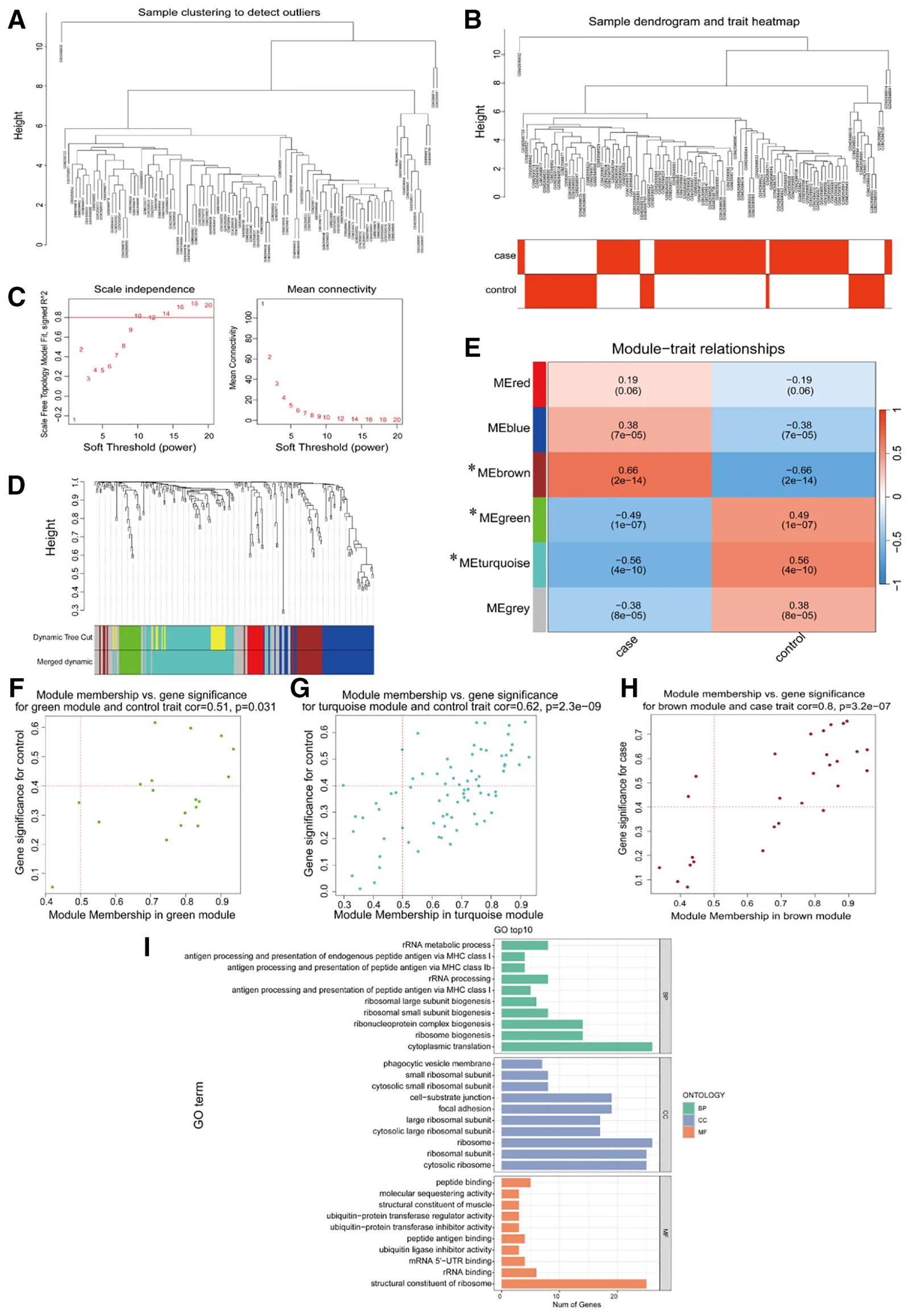
WGCNA network construction and hub gene identification. (A) Sample clustering dendrogram based on gene expression profiles. No significant outliers were observed among 104 samples (69 AS, 35 controls), indicating high data quality. (B) Sample correlation heatmap showing inter-sample Pearson correlation. The AS group (red) and control group (white) are annotated below. (C) Soft-threshold power selection: left, scale-free topology fitting index (*R*^2^) versus power; right, mean connectivity versus power. The optimal soft threshold was chosen at power = 16 (*R*^2^ = 0.867). (D) Module clustering and merging: upper, preliminary modules identified by dynamic tree cut; lower, merging of similar modules resulted in 6 final modules. (E) Module-phenotype correlation heatmap: red indicates positive correlation; blue indicates negative. Significant modules: green (*r* = 0.49, *P* = 1e−07), turquoise (*r* = 0.56, *P* = 4e−10), brown (*r* = 0.66, *P* = 2e−14). (F–H) GS–MM scatter plots of significant modules: F: green; G: turquoise; H: brown. Hub genes are located in the region where GS > 0.4 and MM > 0.5. (I) GO enrichment analysis (top 10 terms) of green, turquoise, and brown modules: BP (green), CC (blue), MF (orange). AS = atherosclerosis, BP = biological processes, CC = cellular components, GO = gene ontology, MF = molecular functions, MM = module membership, WGCNA = weighted gene co-expression network analysis.

Based on the extensive transcriptome abnormalities identified above, we further explored the co-expression patterns of AS-related genes through WGCNA to identify functional modules that are closely related to disease phenotypes. No abnormal samples were detected through clustering analyses, and the correlation heat map between samples confirmed the reliability of the data (Figs. 2A and 2B). The optimal soft threshold was determined to be 16 (Scale-Free Topology Model Fit *R*^2^ = 0.867, mean connectivity = 7.48, Fig. 2C) by screening for scale-free network characteristics, and a co-expression network was constructed based on it.

The gene modules were identified using a dynamic shear tree method, and 6 co-expression modules were finally obtained after merging (Fig. 2D). Subsequently, 3 modules were significantly associated with AS through module-phenotypic correlation analysis: the green module (*r* = 0.49, *P* = 1e−07), the turquoise module (*r* = 0.56, *P* = 4e−10), and the brown module (*r* = 0.66, *P* = 2e−14) (Fig. 2E). Fifty-four hub genes were screened from these 3 modules in combination with gene significance (> 0.4) and module membership (> 0.5) (Figs. 2F–2H). GO functional enrichment analyses showed that these hub genes closely participated in BP such as ribosomal RNA metabolism and antigen presentation (Fig. 2I), which is consistent with the activation trend of immune-related pathways in Section 3.1, suggesting that these modules may participate in the AS process by regulating immune function.

### 3.3. Identification and functional annotation of core hub genes

**Figure 3.**
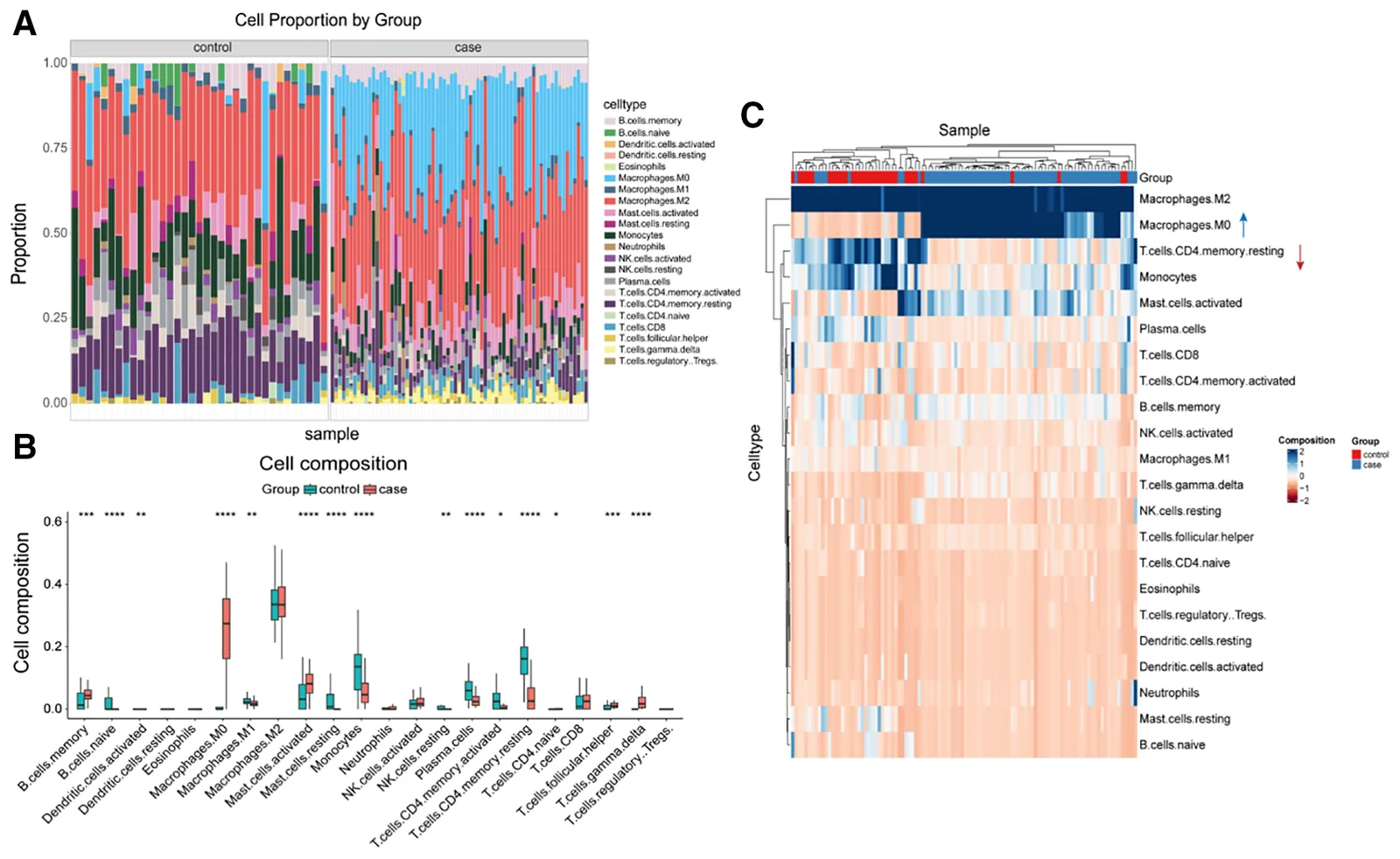
Screening and functional analysis of disease-associated core genes. (A) The Venn diagram identifies 12 core genes at the intersection of 2596 DEGs (green) and 54 hub genes (red). (B) GO enrichment of these 12 core genes reveals MHC class I antigen presentation as the top pathway (FDR < 0.001; bubble size = gene count, color = FDR), prominently involving HLA-G, HLA-A, HLA-C, and B2M. (C) Violin plots confirm significant upregulation of these MHC class I components in disease (red) versus controls (blue) (***P < .001 by *t*-test). B2M = beta-2-microglobulin, DEG = differentially expressed genes, FDR = false discovery rate, GO = gene ontology, HLA-A = major histocompatibility complex, class I, A, HLA-C = major histocompatibility complex, class I, C, HLA-G = major histocompatibility complex, class I, G, MHC = major histocompatibility complex.

To identify core genes with both differential expression patterns and central regulatory roles, we intersected the DEGs identified in Section 3.1 with the hub genes from Section 3.2. This analysis yielded 12 core genes: G protein subunit alpha i2, HLA-G, ATPase H+ transporting V0 subunit c, HLA-A, glutathione peroxidase 1, HLA-C, beta-2-microglobulin (B2M), tropomyosin 2, transgelin, heat shock protein family B (small) member 1, myosin heavy chain 11, and matrix Gla protein (Fig. 3A). GO functional enrichment analysis further revealed that these core genes were significantly enriched in pathways related to “antigen processing and presentation.” Among these, the pathway with the highest enrichment score was “antigen processing and presentation of peptide antigen via MHC class Ib” (Fig. 3B), involving the coordinated participation of HLA-G, HLA-A, HLA-C, and B2M.

Expression validation demonstrated that these 4 genes were significantly upregulated in the AS group compared to healthy controls (***P < .001) (Fig. 3C).

These findings indicate that HLA-G, HLA-A, HLA-C, and B2M are not only differentially expressed but also occupy key regulatory positions within the co-expression network, implicating them as pivotal components of immune regulatory pathways in AS.

Given their central role in antigen presentation and immune modulation, these genes may serve as potential molecular biomarkers for AS. Additionally, their involvement in immune response regulation suggests a possible link to plaque stability and the associated risk of thrombosis.

### 3.4. Immune microenvironment dysregulation and thrombosis risk in AS

Functional enrichment analyses from Sections 3.1 and 3.3 consistently indicated significant activation of immune-related pathways in AS. To further characterize the immune microenvironment in AS, we performed immune cell infiltration analysis.

**Figure 4.**
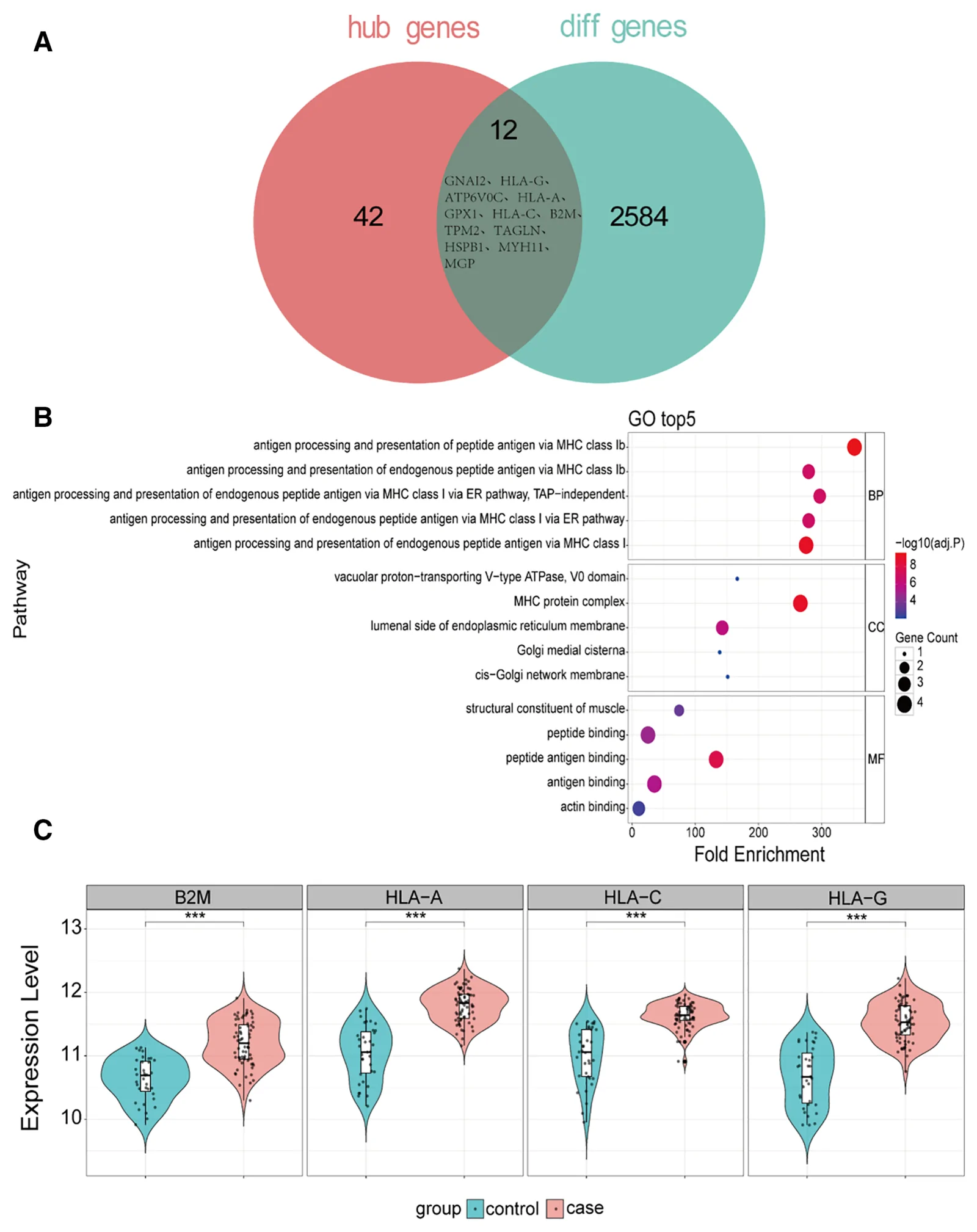
Immune cell composition differences between AS and control groups. (A) Stacked barplot of 22 immune cell types per sample. Compared to controls (left), AS samples (right) show increased M0 macrophages (blue). (B) Boxplot comparison of immune cell proportions between AS (red) and control (blue) groups; significant differences observed in M0 macrophages and resting CD4^+^ memory T cells (****P < .0001, Wilcoxon test). (C) Heatmap of immune cell abundance across samples (rows) and cell types (columns). Blue indicates high abundance, red indicates low. Significant differences highlighted: M0 macrophages (blue arrow) and CD4^+^ memory T cells (red arrow). AS = atherosclerosis, CD = cluster of differentiation, ER = endoplasmic reticulum, HLA-A = major histocompatibility complex, class I, A, HLA-C = major histocompatibility complex, class I, C, HLA-G = major histocompatibility complex, class I, G, MHC = major histocompatibility complex, TAP = transporter associated with antigen processing.

Using the Cell-type Identification By Estimating Relative Subsets Of RNA Transcripts algorithm with the leukocyte signature matrix, we quantified the relative proportions of 22 immune cell subsets in AS and healthy control samples. The results revealed significant differences in immune cell composition between the 2 groups (Fig. 4A). Specifically, box plot analysis demonstrated a marked increase in the proportion of M0 macrophages in AS samples compared to controls (*P* < .0001), while the proportion of resting CD4^+^ memory T cells was significantly reduced (*P* < .0001) (Fig. 4B). A heatmap visualization further illustrated these differences in immune cell abundances between groups (Fig. 4C). These findings align with the immune-related pathway dysregulation observed in Section 3.1, particularly in antigen processing and presentation pathways. The increased infiltration of M0 macrophages and the depletion of resting CD4^+^ memory T cells are indicative of immune microenvironment dysregulation in AS, which may contribute to disease progression. Moreover, this immune imbalance may enhance inflammatory responses and procoagulant activity, thereby promoting thrombus formation following plaque rupture and increasing the risk of acute vascular events.

### 3.5. Gene-immune cell correlation and diagnostic value

**Figure 5.**
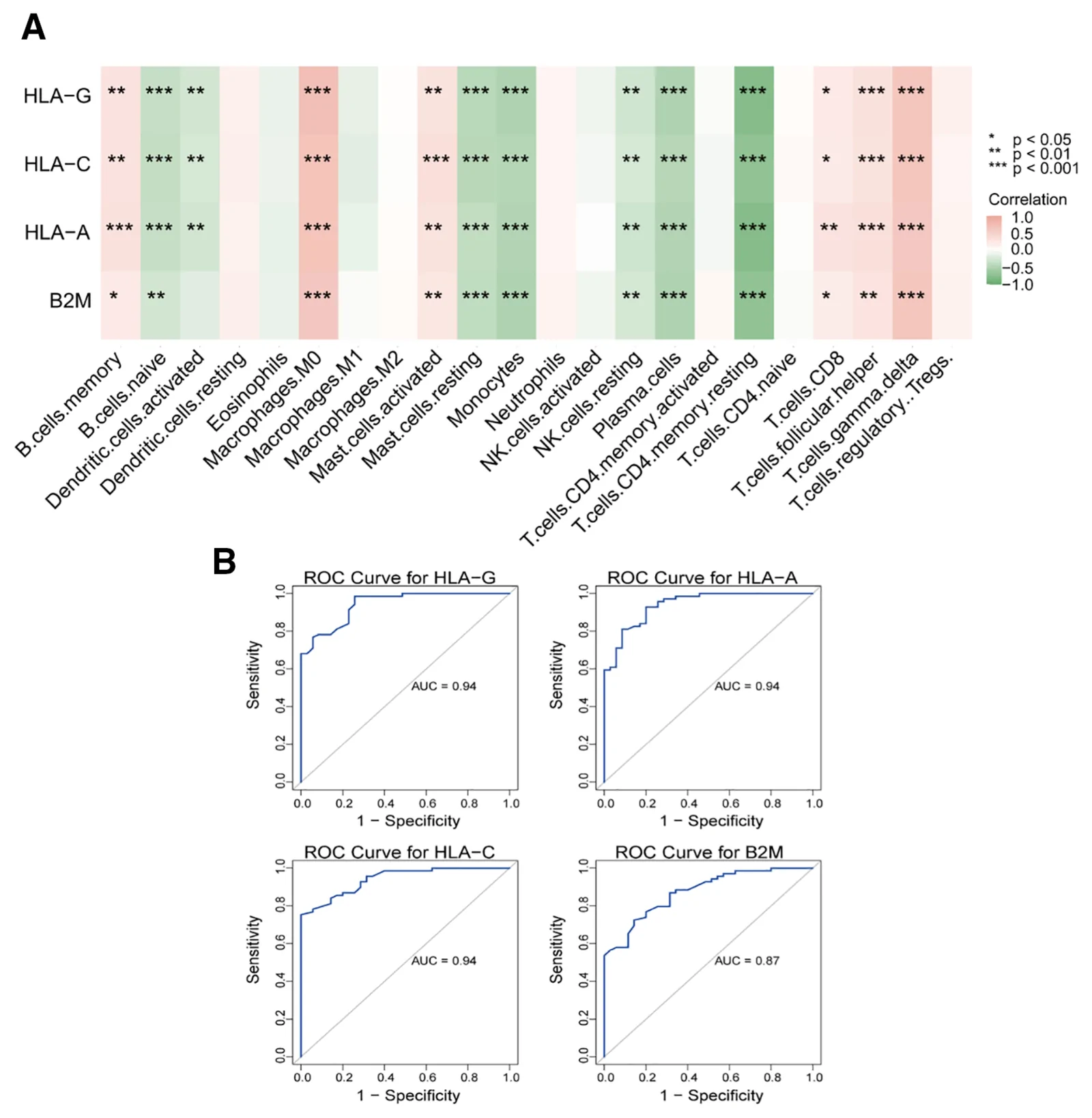
Gene-immune cell correlation and diagnostic value in AS. (A) Heatmap of Spearman correlations between core genes (HLA-G, HLA-A, HLA-C, B2M) and immune cell proportions. Red indicates positive correlation, green indicates negative. Significance: *P < .05, **P < .01, ***P < .001 (FDR-adjusted). (B) ROC curves evaluating the diagnostic efficacy of core genes for AS. AUCs: HLA-G = 0.94, HLA-A = 0.94, HLA-C = 0.94, B2M = 0.87. The gray dashed line represents a random classifier (AUC = 0.5). AS = atherosclerosis, AUC = area under the curve, B2M = beta-2-microglobulin, FDR = false discovery rate, HLA-A = major histocompatibility complex, class I, A, HLA-C = major histocompatibility complex, class I, C, HLA-G = major histocompatibility complex, class I, G, ROC = receiver operating characteristic.

To elucidate the relationship between the core genes identified in Section 3.3 and the immune cell alterations described in Section 3.4, as well as to assess their clinical diagnostic potential, we performed correlation analyses and diagnostic efficacy evaluations. Spearman rank correlation analysis showed that the 4 core genes HLA-G, HLA-A, HLA-C, and B2M were significantly positively correlated with the proportion of macrophage M0 (*P* < .001) and significantly negatively correlated with the proportion of resting CD4^+^ memory T cells (*P* < .001, Fig. 5A). These findings directly link core gene expression to the immune microenvironmental characteristics of AS, suggesting a potential role in modulating macrophage and T cell-mediated immunopathology. Furthermore, ROC curve analysis demonstrated excellent diagnostic performance, with HLA-G, HLA-A, and HLA-C exhibiting AUC values of 0.94, and B2M an AUC of 0.87 (Fig. 5B). These results indicate that the 4 genes possess high diagnostic accuracy within this cohort in distinguishing AS patients from healthy controls. Collectively, these findings suggest that the identified core genes not only regulate immune responses and influence thrombosis risk but also serve as promising diagnostic biomarkers for AS.

## 4. Discussion

In this study, we identified 4 core genes (HLA-G, HLA-A, HLA-C, and B2M) through comprehensive bioinformatics analyses. These genes are implicated in the dysregulation of the immune microenvironment in AS, potentially mediating their effects by modulating the functions of M0 macrophages and resting CD4^+^ memory T cells. This immune imbalance is closely associated with an increased risk of thrombosis. Our findings provide important mechanistic insights into the immunopathology of AS and offer a theoretical foundation for the development of novel strategies aimed at thrombosis prevention and AS management.

At the transcriptome level, a total of 2596 DEGs were identified in the arterial tissues of patients with AS, which are significantly enriched in immune-related pathways including “antigen processing and presentation” and “chemokine signaling pathway.” These findings are consistent with previous studies, which have demonstrated that immune activation and inflammatory signaling play pivotal roles in the pathogenesis of AS. AS is recognized as a chronic inflammatory disease, wherein immune activation plays a central role in plaque formation and thrombosis. Following endothelial injury, ox-LDL acts as a key antigen and is processed by antigen-presenting cells, which present it to T lymphocytes via MHC molecules, thereby initiating an adaptive immune response.^[12]^ In this study, we found that DEGs were significantly enriched in the MHC class I molecule-mediated antigen presentation pathway, suggesting that this pathway may contribute to the progression of AS by activating pathogenic T cells, particularly CD8^+^ cytotoxic T lymphocyte. Activated CD8^+^ T cells can release perforin and granzyme B, inducing apoptosis of vascular cells, which in turn compromises fibrous cap integrity and increases the risk of plaque rupture.^[13]^ It is worth noting that the activation of the “chemokine signaling pathway” may further amplify the local inflammatory response. Previous studies have demonstrated that chemokines play a crucial role in recruiting additional monocytes and T lymphocytes into atherosclerotic plaques, thereby accelerating lipid core expansion and promoting fibrous cap degradation, which collectively exacerbate plaque instability.^[14]^ Following plaque rupture, exposed procoagulant factors (such as tissue factor) interact with activated platelets, leading to the upregulation of P-selectin on the platelet surface. Through binding with P-selectin glycoprotein ligand-1 on leukocytes, this interaction facilitates leukocyte recruitment and adhesion at the site of injury. Additionally, activated platelets release chemokines, including C-C motif chemokine ligand 5 and platelet factor 4, which further amplify local inflammatory and procoagulant responses. This cascade is closely linked to pro-inflammatory cytokines such as TNF-α secreted by immune cells, thereby perpetuating the inflammatory–thrombotic cycle within atherosclerotic lesions.^[15]^ Therefore, the abnormal activation of immune-related pathways identified in this study may be a key molecular mechanism underlying the progression of AS from chronic inflammation to thrombotic complications. By overlapping the DEGs with hub genes identified through WGCNA, we screened 12 core genes, among which HLA-G, HLA-A, and HLA-C belong to the MHC class I family. These genes, along with B2M, were co-enriched in the “antigen processing and presentation of peptide antigen via MHC class Ib” pathway and were found to be significantly upregulated in the AS group. MHC class I molecules play a pivotal role in presenting antigenic peptides derived from endogenous sources, such as ox-LDL-modified peptides, to CD8^+^ T cells, thereby initiating cytotoxic immune responses.^[16]^ HLA-A and HLA-C, as classical MHC class I molecules, were found to be significantly upregulated in the arterial tissues of AS patients (Fig. 3C), indicating heightened antigen-presenting activity within atherosclerotic plaques. This enhanced presentation may potentiate vascular endothelial injury and foam cell formation by promoting the activation of CD8^+^ cytotoxic T lymphocytes. In contrast, HLA-G, a nonclassical MHC class Ib molecule, is traditionally recognized for its immunosuppressive functions, including the inhibition of natural killer cell cytotoxicity and T cell-mediated immune responses.^[17]^ However, recent studies have indicated that HLA-G may contribute to immune imbalance in AS through its abnormally elevated expression. It has been reported that HLA-G mediates negative feedback immunomodulation via monocyte surface expression, leading to the downregulation of Th1 and Th2 cytokine production, thereby modulating the inflammatory response. Additionally, in vitro studies have demonstrated that soluble HLA-G can inhibit endothelial cell proliferation and migration, as well as regulate endothelial activity, suggesting a potential role in maintaining vascular homeostasis. These findings imply that aberrant HLA-G expression may influence both immune regulation and endothelial function, which are intricately linked to the pathogenesis of AS.^[18]^ In this study, the elevated expression of HLA-G observed in the AS group (Fig. 3C) may represent a “double-edged sword” in the progression of AS. On one hand, HLA-G-mediated immunosuppression could attenuate excessive immune activation within plaques, thereby reducing the risk of acute plaque rupture. On the other hand, overactive immunoinhibition may impair the clearance of dysfunctional or apoptotic cells within the lesion, promoting plaque persistence and chronic inflammation, which could eventually contribute to plaque calcification and increased lesion stability at the cost of long-term vascular health. This dualistic role highlights the complexity of immune regulatory mechanisms in AS and underscores the need for a balanced immune response in maintaining plaque homeostasis.^[19]^ This dual role may be key to the balance of the AS immune regulatory network. As an important part of MHC class I molecules, B2M is essential for its folding, transport, and antigen presentation functions, allowing it to stabilize the membrane positioning of HLA-A/C, enhance the efficiency of antigen presentation, and exacerbate T cell-mediated inflammation.^[20]^ Together, these mechanisms suggest that the human leukocyte antigen family and B2M may be key molecules that promote immune imbalance and plaque instability by regulating antigen presentation and immune cell function.

Immunoinfiltration analysis revealed a marked increase in the proportion of M0 macrophages and a significant reduction in resting CD4^+^ memory T cells in AS patients. Furthermore, the abundance of these immune cell populations exhibited a strong correlation with the expression levels of the core genes HLA-G, HLA-A, HLA-C, and B2M, suggesting that these genes may play a pivotal role in modulating the immune microenvironment associated with AS progression. These findings offer a novel perspective on the intricate relationship between the immune microenvironment in AS and thrombotic risk. As unpolarized precursor cells, M0 macrophages can be driven towards a pro-inflammatory M1 phenotype under the influence of ox-LDL and inflammatory mediators present within the AS milieu. Activated M1 macrophages contribute to plaque destabilization by secreting matrix metalloproteinases, which degrade fibrous cap collagen, and by producing cytokines such as TNF-α that induce apoptosis of vascular smooth muscle cells. This cascade of events leads to fibrous cap thinning, rendering plaques more vulnerable to rupture and precipitating acute thrombotic complications.^[14]^ In this study, core genes, including members of the human leukocyte antigen family and B2M, were found to be positively correlated with macrophage M0 infiltration, suggesting their potential involvement in plaque instability through the regulation of macrophage function. Conversely, a significant reduction in resting CD4^+^ memory T cells may compromise immunomodulatory mechanisms within the atherosclerotic microenvironment. Under physiological conditions, resting CD4^+^ memory T cells contribute to immune homeostasis by differentiating into Tregs, which secrete anti-inflammatory cytokines such as interleukin-10, thereby mitigating excessive inflammatory responses. The observed decrease in these T cell subsets may thus impair the anti-inflammatory feedback loop, exacerbating local inflammation and promoting plaque vulnerability.^[21]^ The reduction in the proportion of resting CD4^+^ memory T cells may disrupt the Treg/Th1 balance, resulting in the excessive release of pro-inflammatory cytokines. This dysregulation not only intensifies the inflammatory milieu within atherosclerotic plaques but may also contribute to platelet hyperactivation and the amplification of thrombin generation. Consequently, this creates a vicious cycle wherein inflammation and thrombosis mutually reinforce each other, accelerating plaque progression and increasing the risk of acute thrombotic events.^[22]^ In this study, the expression of core genes demonstrated a significant negative correlation with resting CD4^+^ memory T cells, indicating that these genes may indirectly contribute to thrombosis by inhibiting the expansion or activation of this immunomodulatory T cell subset. Furthermore, ROC curve analysis revealed that HLA-G, HLA-A, and HLA-C exhibited excellent diagnostic performance (AUC = 0.94), while B2M also showed high diagnostic efficacy (AUC = 0.87), underscoring their potential as molecular biomarkers for AS. Currently, the diagnosis of AS predominantly relies on imaging modalities such as magnetic resonance imaging, positron emission tomography/computed tomography, and single-photon emission computed tomography, as well as conventional risk factors including dyslipidemia and hypertension; however, there remains a lack of specific molecular markers that can facilitate early detection and risk stratification. The identification of these core genes as candidate biomarkers may thus provide novel tools for the molecular diagnosis and therapeutic targeting of AS.^[23]^

The expression levels of the core genes identified in this study, whether in circulating blood or arterial tissues, may offer valuable molecular insights for the early diagnosis of AS. More importantly, their close association with immune cell infiltration patterns and plaque stability suggests that these genes could serve as potential biomarkers for assessing thrombotic risk, thereby providing a novel molecular basis for both risk stratification and therapeutic targeting in AS.

Despite these findings, our study has limitations. The analysis was performed on a single dataset (GSE100927) with a relatively modest sample size. Although our internal ROC analysis showed promising diagnostic value for the core genes, future validation in larger, independent cohorts is essential to confirm their generalizability as robust biomarkers for AS. Furthermore, the lack of critical clinical metadata, such as patient age, prevents us from assessing its potential confounding effect on our analyses.

Taken together, this study identified HLA-G, HLA-A, HLA-C, and B2M as key regulators of immune microenvironment dysregulation in AS, primarily through their roles in antigen presentation and modulation of macrophage and T cell functions. These molecules may serve as critical links between plaque instability and thrombotic events. The findings not only provide novel insights into the immunopathological mechanisms underlying AS progression but also offer a theoretical foundation for the development of molecular biomarkers for early diagnosis and thrombotic risk assessment in AS.

## Author contributions

**Conceptualization:** Jingping Ge.

**Data curation:** Jingping Ge.

**Formal analysis:** Ke Ma, Jingping Ge.

**Investigation:** Ke Ma.

**Resources:** Jingping Ge.

**Visualization:** Ke Ma.

**Writing – original draft:** Ke Ma.

**Writing – review & editing:** Jingping Ge.


